# Incentives for Reporting Disease Outbreaks

**DOI:** 10.1371/journal.pone.0090290

**Published:** 2014-03-06

**Authors:** Ramanan Laxminarayan, Julian Reif, Anup Malani

**Affiliations:** 1 Center for Disease Dynamics, Economics & Policy, Washington, D. C., United States of America; 2 Princeton University, Princeton, New Jersey, United States of America; 3 Public Health Foundation of India, ISID Campus, Institutional Area, Vasant Kunj, New Delhi, India; 4 Law School and Pritzker School of Medicine, University of Chicago, Chicago, Illinois, United States of America; 5 Department of Finance and Institute of Government and Public Affairs, University of Illinois at Urbana-Champaign, Champaign, Illinois, United States of America; National Institutes of Health, United States of America

## Abstract

**Background:**

Countries face conflicting incentives to report infectious disease outbreaks. Reports of outbreaks can prompt other countries to impose trade and travel restrictions, which has the potential to discourage reporting. However, reports can also bring medical assistance to contain the outbreak, including access to vaccines.

**Methods:**

We compiled data on reports of meningococcal meningitis to the World Health Organization (WHO) from 54 African countries between 1966 and 2002, a period is marked by two events: first, a large outbreak reported from many countries in 1987 associated with the Hajj that resulted in more stringent requirements for meningitis vaccination among pilgrims; and second, another large outbreak in Sub-Saharan Africa in 1996 that led to a new international mechanism to supply vaccines to countries reporting a meningitis outbreak. We used fixed-effects regression modeling to statistically estimate the effect of external forcing events on the number of countries reporting cases of meningitis to WHO.

**Findings:**

We find that the Hajj vaccination requirements started in 1988 were associated with reduced reporting, especially among countries with relatively fewer cases reported between 1966 and 1979. After the vaccine provision mechanism was in place in 1996, reporting among countries that had previously not reported meningitis outbreaks increased.

**Interpretation:**

These results indicate that countries may respond to changing incentives to report outbreaks when they can do so. In the long term, these incentives are likely to be more important than surveillance assistance in prompt reporting of outbreaks.

## Introduction

Although international health regulations require countries to report infectious disease outbreaks, [1] countries face disincentives to do so, including reduced trade and tourism. [2] Donor assistance for surveillance cannot overcome these disincentives, but policies aimed at containing outbreaks, such as providing subsidized vaccines to countries that report outbreaks, could incentivize surveillance and reporting. [2] Here we look at reporting of bacterial meningitis and find evidence that incentives do matter.

Bacterial meningitis caused by *Neisseria meningitides* is the leading cause of meningitis worldwide and a significant global health challenge, especially in sub-Saharan Africa. Meningococcal meningitis epidemics in sub-Saharan Africa recur every 5–12 years and cause about 3,000–10,000 deaths each year. [3] At least 32 meningitis outbreaks were reported globally between 1971 and 2000, including a 1987 outbreak during the Hajj, the annual Muslim pilgrimage to Mecca and Medina in Saudi Arabia, and a 1996 outbreak in Sub-Saharan Africa. [3]–[6]

In response to the 1987 outbreak, Saudi Arabia mandated compulsory bivalent A and C vaccines for all pilgrims, implemented annual vaccination campaigns for all local populations in high-risk areas, and provided oral ciprofloxacin to travelers from the meningitis belt in sub-Saharan Africa in order to lower carriage rates. Despite the vaccination requirements, many pilgrims gained entry without being vaccinated and moreover, these requirements were not strictly enforced. [7], [8] At $55 in 1987, the bivalent meningococcal vaccine was too expensive for many travelers from endemic countries. In fact, small outbreaks of meningococcal disease due to *N. meningitidis* serogroup A were reported from Mecca and Jeddah in 1988 and 1992. [7], [9] Saudi authorities reportedly focused on travelers from countries with endemic meningococcal disease,^7^ and countries sending pilgrims to the Hajj may have been reluctant to report outbreaks lest their citizens be targeted.

In response to the 1996 outbreak, the World Health Organization (WHO) formed the International Coordinating Group (ICG) on Vaccine Provision for Epidemic Meningitis Control to provide subsidized meningococcal vaccines to countries showing that the number of cases per week in affected districts crossed the epidemic threshold. Because vaccine provision is contingent on reporting, countries have an incentive to report promptly. ICG has accelerated improvements in the surveillance system in African countries, which now have incentives to report cases. [10] To date, close to 30 million doses of meningococcal vaccine have been channeled through ICG.

Similar efforts to coordinate the stockpiling and distribution of yellow fever vaccine for Latin America and Africa through the Global Alliance for Vaccines and Immunisation have had a beneficial effect on yellow fever reporting. Similarly, the availability a rinderpest vaccine resulted in improved surveillance and reporting of cases. [11] Countries may explicitly demand access to vaccines as a condition of reporting and sharing biological samples, as was the case with H5N1 samples in Indonesia in 2008. [12]

In this paper, we empirically estimated the effect of the 1988 Hajj vaccination requirement and the ICG vaccine provision on the likelihood that countries would report an outbreak, which we define as reporting a positive number of meningitis cases. Reporting of measles cases was used as a control for time-variant changes in outbreak reporting incentives. We tested two hypotheses. The first is that the Hajj vaccination requirements would depress reporting. The second hypothesis is that the introduction of ICG-supplied vaccines would improve meningitis reporting in countries.

## Materials and Methods

We obtained data on the number of meningitis cases reported by 54 African countries during 1966–2002 and measles cases reported by these same countries during 1980–2002. [13] (A list of these countries is provided in Table S1.) Data on population size and religion were obtained from CIA Fact book. [14] The number of countries reporting is defined as the number reporting a positive number of cases. Data on number of countries reporting and cases reported were categorized into three periods: period one (1980–1987, i.e., before the Saudi vaccination requirements), period two (1989–1995, i.e., from the Saudi vaccination requirement to the ICG vaccination program), and period three (1997–2002, i.e., with the start of the ICG vaccination program).

We drop transition years between policy regimes in our statistical analyses because policy regimes start midyear. The Hajj vaccination requirement started some time in 1988, so we drop that year. Although the ICG mechanism was formally announced in 1997, the mechanism was actually begun in 1996 (personal communication with David Heymann, former Executive Director of the Communicable Disease Cluster at WHO, March 12, 2011). Therefore, we drop 1996 as well. Finally, the ICG had its final meeting in 2003, when it was replaced with a different vaccination distribution mechanism. Therefore, we drop 2003 from the analysis.

To test our two hypotheses, that the Saudi requirements reduced reporting in period two and that the ICG program increased reporting in period three, we construct a measure of the trend in disease reporting. First, we created a binary variable that indicates whether a country reported a positive number of meningitis cases in a given year. We first-differenced this variable to measure the change in reporting over time for a country. The mean of this first difference in any given year measures the year-to-year change in the fraction of African countries reporting meningitis outbreaks that year. We constructed a similar variable for measles reporting. We intend to employ trends in reporting of measles to serve as a control for unobservable influence on health and reporting by countries.

**Figure 1:**
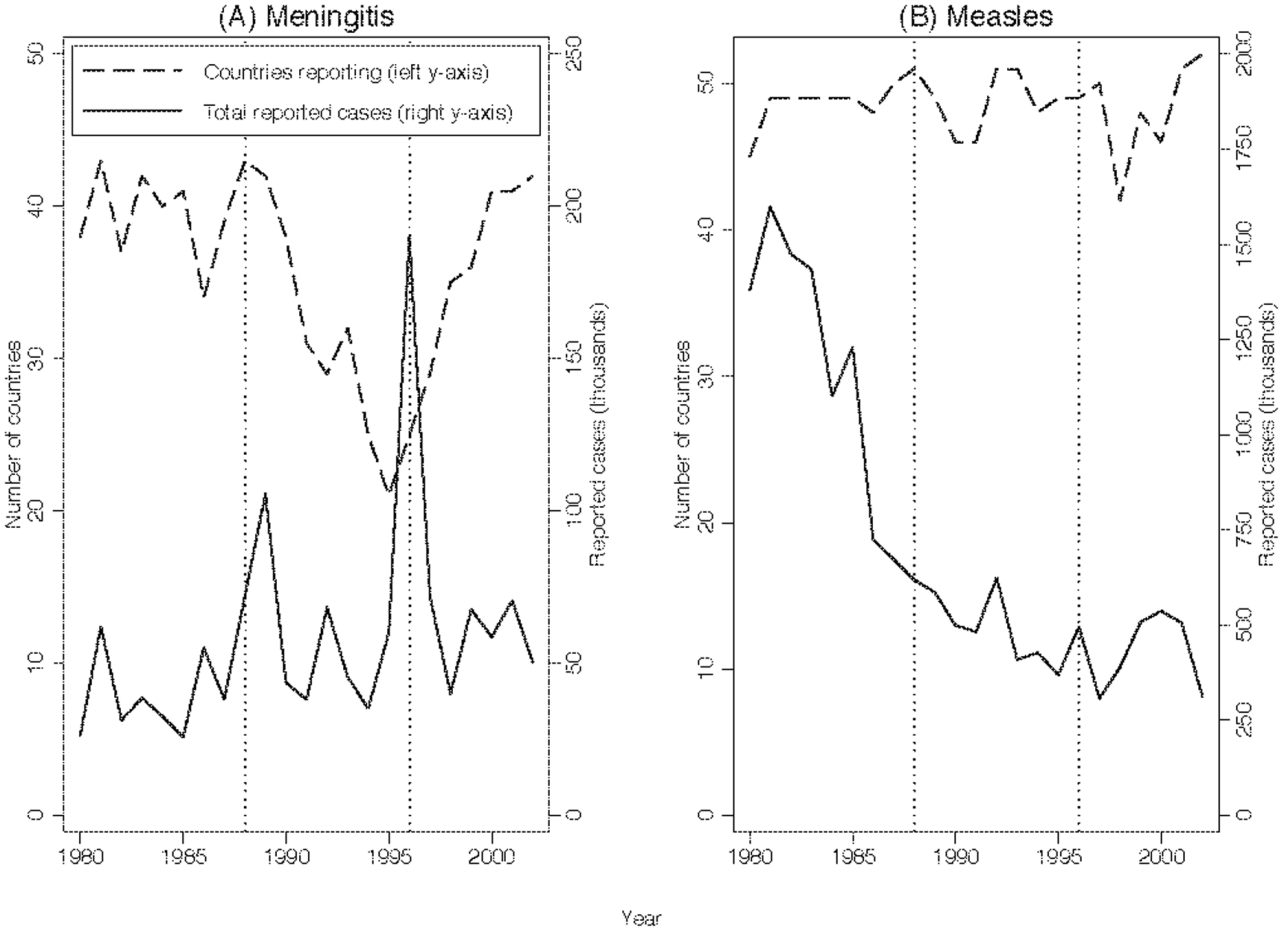
Meningitis and measles reporting by 54 countries in Africa, 1980–2002.

The primary test of our two hypotheses is presented in Figure 1. The dashed line in Panel A plots the number of countries that report a positive number of meningitis cases during 1980–2002. The range of data is restricted to focus on dates that the data on meningitis cases, the data on measles cases, and the policy treatment periods overlap. The solid line in the panel reports the total number of meningitis cases reported each year. Vertical dotted lines indicate the start of the Hajj vaccination requirement (1988) and the start of the ICG subsidized vaccine program (1996). The number of countries that reported outbreaks peaks in 1987 and then falls dramatically after 1988. The decline ends in 1995 and continues to spike upwards after 1996, when the ICG program counteracted the disincentive imposed by the Hajj vaccination requirement. In contrast, the number of countries reporting a positive number of measles cases is roughly the same before the Hajj vaccination requirement as after it (Panel B). Moreover, this number appears to have slightly decreased for a few years after the ICG program began in 1996.

To determine whether the trends reported in Figure 1 were statistically significant, we conducted a Wilcoxon rank-sum test to determine whether the distribution of changes in year-to-year reporting status of countries differs across the three periods. For meningitis reporting, we find that reporting changes during the Hajj requirement are lower than those in the pre-Hajj requirement period (p = 0·09), though insignificant, and trends during the ICG program are significantly higher than in prior periods (p = 0·04 versus pre-Hajj, p<0·01 versus Hajj). By contrast, reporting trends for measles are highly insignificantly different across the three policy periods.

To control for underlying country-level reporting, we estimated a linear regression on country-level data on meningitis reporting during 1980–2002. The dependent variable is the change in the year-to-year reporting status of countries in a given year. Independent variables include indicators for the Hajj vaccination requirement period (1989–1995) and ICG program period (1997–2002) to capture the influence of these policies on reporting, and country fixed effects to capture invariant, country-level unobservable variables. Standard errors are clustered at the country-level to allow correlation between idiosyncratic differences in country reporting over time. We ran five versions of this regression. In specification (1), we examined only meningitis reporting. In specification (2), we examined only measles reporting. In specification (3), we examined both meningitis and measles reporting. To separate the influence of policy treatments on each disease, however, we included interactions between the policy variables and the type of disease being reported. Specification (4) examines meningitis reporting but limits the sample to Muslim countries. Specification (5) also examines meningitis reporting but limits the sample to non-Muslim countries.

## Results

Regression results are presented in Table 1. Before the Hajj vaccination requirement, there was a slight negative trend in meningitis reporting. Relative to this baseline trend, meningitis reporting fell after the Hajj vaccination requirement. According to specification (1), each year an additional 5.0% (p = 0·02) of countries stopped reporting a positive number of meningitis cases. After the ICG program, however, reporting began trending upwards, with 6.2% (p = 0·02) more countries reporting each year. According to specification (2), reporting of measles outbreaks does not show similar, statistically significant trend breaks under the Hajj requirement or the ICB program. Specification (3) uses the measles reporting trends as a control for meningitis reporting trends. The comparison mitigates the decline in meningitis reporting relative to baseline under the Hajj requirement and amplifies the increase in reporting under the ICG program. The change in trends under the ICG regime, however, remain statistically significant. (We also performed the analysis using cholera instead of measles as a control. Those results, presented in Figure S1 and Table S2, are similar.) Comparing specification (4) to specification (5) shows that the estimated effect of the Hajj vaccination requirement is larger in Muslim than in non-Muslim countries, as expected.

**Table 1:** Regression analysis of reporting trends, 1980–2002.

	(1)	(2)	(3)	(4)	(5)
	Meningitis	Measles	Meningitis relative to measles	Meningitis: Muslim countries	Meningitis: non-Muslim countries
Baseline	−0.001	0.007	0.008	0.003	0.018
	(0.008)	(0.006)	(0.007)	(0.009)	(0.024)
Hajj policy	−0.050***	−0.016	−0.034*	−0.054***	−0.024
	(0.017)	(0.013)	(0.021)	(0.017)	(0.059)
ICG	0.062***	−0.004	0.066***	0.053***	0.135***
	(0.018)	(0.010)	(0.020)	(0.019)	(0.050)
Obs.	1,080	1,080	2,160	960	120

Note. Observations are at the country x disease x year level. The data span 1980–2002 but exclude 1988 and 1996, transition years between policy regimes. Dependent variable is the change in an indicator for whether a country reported a disease; so the dependent variable takes values -1, 0, or 1. Specification (1) includes data on meningitis reporting only. Specification (2) includes data on measles reporting only. Specification (3) includes data on both types of reporting. Specifications (1), (2), (4), and (5) include policy period indicators and country fixed effects. Specification (3) includes period indicators, those indicators interacted with a meningitis disease indicator, and country fixed effects. Standard errors are reported below coefficients. ***/**/* indicate p<0•01/0•05/0•1. Specification (4) includes only data for countries with Muslim (>1%) populations. Specification (5) includes only data on non-Muslim countries.

Although the Hajj vaccination requirement reduced the probability that countries report a positive number of meningitis cases (see Figure 1, Panel A), it was not associated with a statistically significant reduction in the total number of meningitis cases reported. A possible explanation is that the Hajj requirement only reduced reporting among countries that previously had small outbreaks – outbreaks with few reported cases. After the Hajj requirement, these countries could more credibly report no outbreak than a country that previously had large outbreaks. If countries with previously large outbreaks stopped reporting any outbreaks, the Saudi government would be unlikely to believe these countries truly had no outbreak.

To test this explanation, we sorted countries into whether they were part of the so-called “meningitis belt”, 21 countries that are historically prone to meningitis outbreaks. According to Table 2, countries in the meningitis belt had four-and-a-half times as many cases, on average, during 1966–1979 as countries outside the belt. Countries inside the meningitis belt were nearly 20% more likely to report an outbreak (a positive number of cases) than those outside the belt. Countries outside the belt had smaller populations. The percentages of their populations that were Muslim were not appreciably lower than countries inside the belt.

**Table 2:** Summary statistics, by whether countries are in meningitis belt.

In menin-gitis belt	Population (2008, mil.)	Muslim population (2008, percent of total population)	Percent of countries reporting any cases (1966–79)	Reported cases per country (1966–79, thous.)
No	12.21	44.08	64.94	0.3
	(17.58)	(40.44)	(30.66)	(0.50)
Yes	26.68	48.57	82.31	1.37
	(34.11)	(31.02)	(21.24)	(1.88)
All African	17.84	46.18	71.69	0.72
countries	(26.15)	(36.22)	(28.57)	(1.33)

Note: Observations are at the country-year level. Means shown. Standard deviations in parentheses.

**Figure 2:**
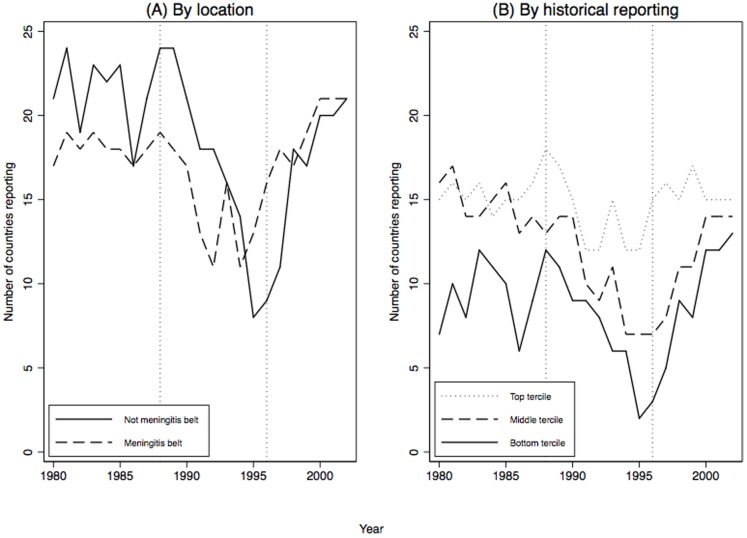
Meningitis reporting in African countries during 1980–2002, by whether countries are in meningitis belt or not.

Figure 2 (Panel A) plots the number of countries inside and outside the meningitis belt that report a meningitis outbreak during 1980–2002. Vertical dotted lines again indicate the start of the Hajj vaccination requirement and the announcement of the ICG vaccine program. The number of countries outside the belt that reported outbreaks fell dramatically after 1988. The drop in reporting for countries inside the belt—countries that could not credibly hide an epidemic given their history of outbreaks—was much lower. There was a sharp spike in reporting rates outside the belt after 1996, when the ICG program counteracted the disincentive imposed by the Hajj vaccination requirement.

Aggregate, annual statistics on the fraction of countries that report a meningitis outbreak ignore a great deal of the dynamics of such outbreaks. For example, outbreaks are seasonal, they may last multiple years, they are affected by climate, and they depend on the local impact of reactive vaccinations. Our annual and country-level data abstract from these important considerations. Because our analysis employs country fixed effects and interprets treatment effects at the annual and national level, many of these considerations are orthogonal to our treatment variables. As a result, they do not bias our estimates of the impact of the Hajj vaccination requirement and the ICG on outbreak reporting. To test whether multi-year outbreaks might affect the analysis, we replicated the regression analysis using two-year differences instead of one-year differences. Our results, presented in Table S3, were not substantively affected.

**Figure 3:**
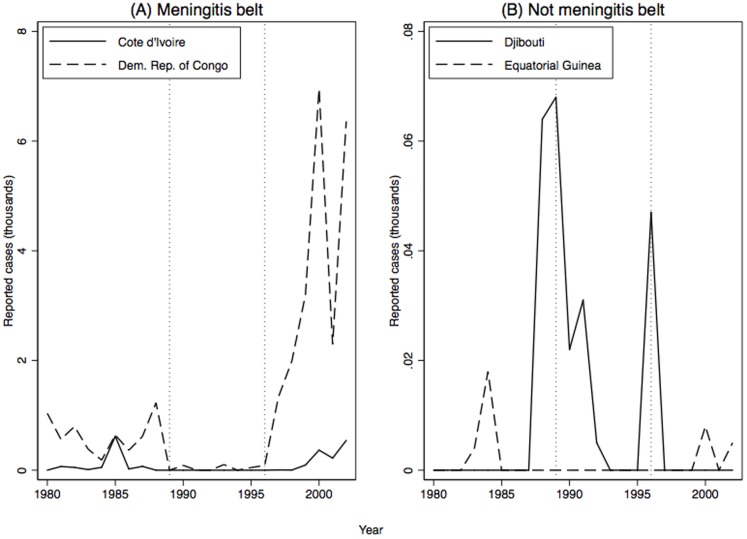
Meningitis reporting in four randomly selected countries, two inside and two outside the meningitis belt.

Nevertheless, it is helpful to look at reported outbreaks in a few specific countries inside and outside the belt to see how their reporting responded to changes in reporting incentives. Figure 3 plots the number of cases reported by four randomly selected countries, two inside the meningitis belt (Cote d'Ivoire and Democratic Republic of Congo) and two outside the meningitis belt (Djibouti and Equatorial Guinea). As expected, far more cases are reported inside the belt countries than outside the belt countries. Although there is a great deal of annual variability from year to year, both sets show a drop in reporting of cases during the Hajj vaccination requirement period and three of the four show an increase in reporting during the ICG program period.

Although our hypotheses concern the probability of reporting an outbreak conditional on an outbreak, our empirical analysis focuses simply on the unconditional probability of reporting a positive number of cases. The unconditional probability of a report is the conditional probability times the probability of an outbreak. If the Hajj vaccination requirement or the ICG program directly affected the unconditional probability of an outbreak and the sign of this effect were similar to the effect on the conditional probability of reporting, our results might incorrectly validate our hypotheses. However, it is unlikely the trends in reporting depicted in Figure 1 are the result of the policy regimes requirements having actually altered vaccination practices and thus the number of meningitis outbreaks.

First, if the Hajj vaccination requirement induced greater vaccination, even countries that historically reported the largest number of cases should have experienced a reduction in the probability of reporting outbreaks. To check if this was the case, we sorted countries into terciles based on the average number of meningitis cases reported from 1966 to 1979. There are 18 countries per tercile. Figure 2 (Panel B) plots the number of countries in each tercile that report a meningitis outbreak during 1980–2002. The number of countries in the bottom two terciles that reported outbreaks fell dramatically after 1988. The drop in reporting among top tercile countries was much lower. Therefore, it is unlikely the Hajj vaccination requirement reduced the actual number of outbreaks and likely that our measure of unconditional probability of reporting tracks the conditional probability of reporting during the requirement period.

Second, if vaccination policies had an effect on the actual number of outbreaks rather than merely on reporting, the ICG program should have led to a reduction in reported outbreaks. Yet Figure 1 and the regression analysis in Table 1 suggest that the ICG program increased the number of reported outbreaks. Therefore, our finding, if anything, understates the extent to which the ICG program increased the conditional probability of reporting.

It should be noted that the establishment of the ICG mechanism was also the occasion for the WHO to develop a standardized methodology of reporting meningitis. This methodology significantly improved reporting, especially in the meningitis belt. This procedural effect is distinct from the effect of a reward in the form of vaccine access. However, the procedural effect of ICG is unlikely to explain all of the change in reporting during period three. Procedural improvements were expected to have larger effects in meningitis belt countries, yet Figure 2 (Panel A) shows a larger effect in non-belt countries (and in bottom tercile countries). Moreover, procedural improvements should have greater impact on the number of cases reported rather than whether a country reports. Our findings demonstrate a bump in the latter.

Finally, although our results support our hypothesis that the Hajj vaccination requirement and the ICG program affected the incentives to report meningitis outbreaks, i.e., a positive number of meningitis cases, we caution that observational studies of this nature cannot prove causality and are subject to unobserved confounding variables that may not be adequately accounted for by our control variables.

## Discussion

Incentives for surveillance and reporting are fundamental for prompt reporting of outbreaks to curtail potential global pandemics. For instance, modeling studies have suggested that an emerging pandemic of avian influenza could be contained if cases suggesting human-to-human transmission are reported within three weeks of the index case. [15], [16] Surveillance and reporting decisions, as well as the initiation of early rapid containment, are the responsibilities of national governments. Although efforts to build a global early warning system have focused on technical assistance for improved detection in countries where disease outbreaks are likely, modern surveillance systems do nothing to improve countries' incentives for reporting.^2^ A country with few incentives to report will not effectively implement surveillance. Moreover, outbreak reporting involves more than sharing data on epidemiological burden—it also involves sharing of biological data.

Here we show that incentives, rather than just financial assistance to build surveillance networks, could alter reporting decisions, especially when the burden of disease is low. The ability to contain outbreaks is also important; countries are unlikely to look for outbreaks that they can do little to contain, especially under threat of sanctions. In our study, policies that changed the benefits of reporting had little effect on reporting by countries with a large burden of meningococcal meningitis, but significantly altered reporting by countries with fewer cases.

## Supporting Information

Figure S1 **Meningitis and cholera reporting by 54 countries in Africa, 1980–2002.**
(TIFF)

Table S1 **List of countries in data sample, by whether they are located in the meningitis belt.**
(TIFF)

Table S2 **Regression analysis comparing meningitis to cholera reporting trends, 1980–2002.**
(TIFF)

Table S3 **Regression analysis of reporting trends, 1980–2002, using two-year differences in reporting status as a measure of trends.**
(TIFF)
