# Supplementary figures and images for: Incentives for Reporting Disease Outbreaks
Source: PLoS One. 2014 Mar 6;9(3):e90290. doi: 10.1371/journal.pone.0090290 (PMC3946072; doi:10.1371/journal.pone.0090290)

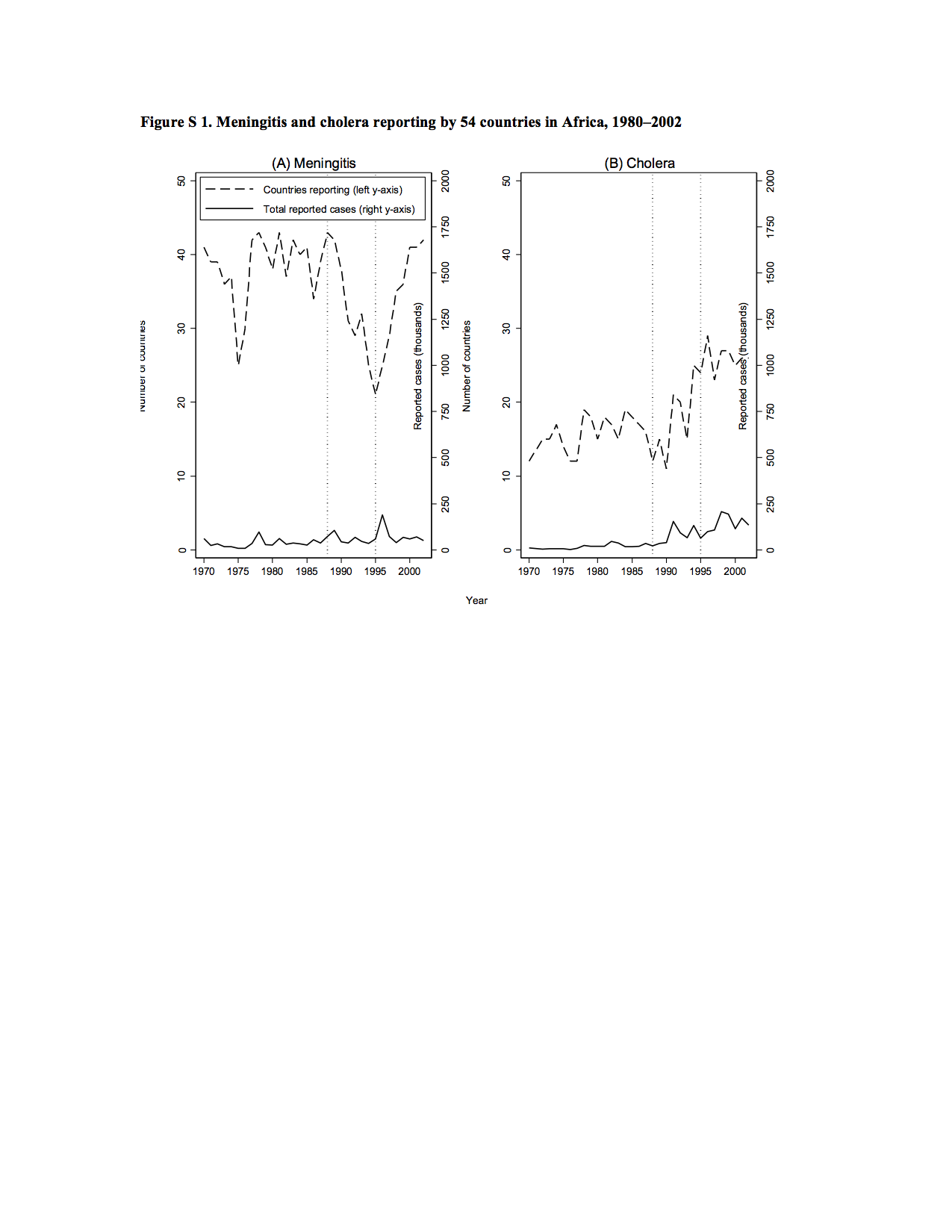

Supplement: Figure S1 — Meningitis and cholera reporting by 54 countries in Africa, 1980–2002. (TIFF) [file pone.0090290.s001.tiff]

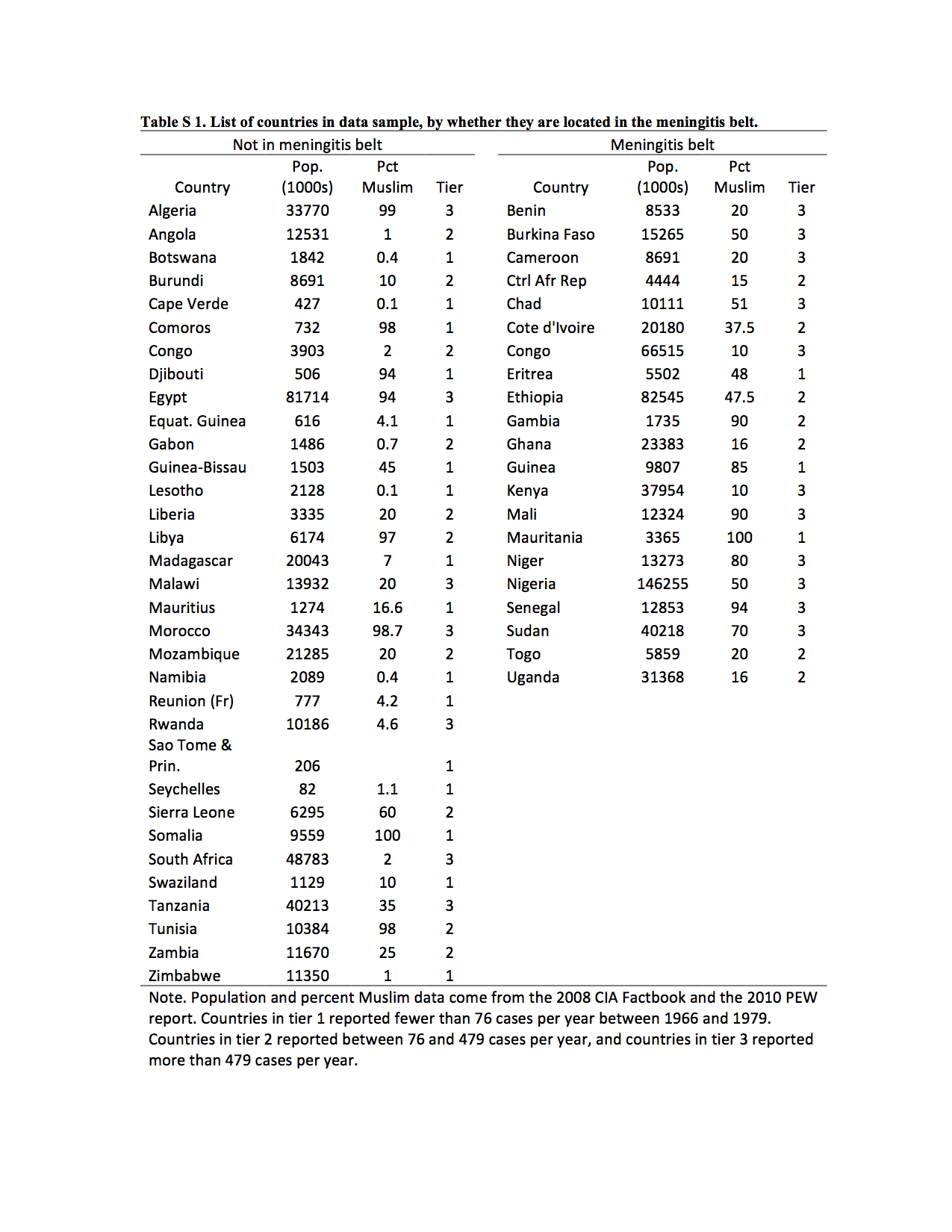

Supplement: Table S1 — List of countries in data sample, by whether they are located in the meningitis belt. (TIFF) [file pone.0090290.s002.tiff]

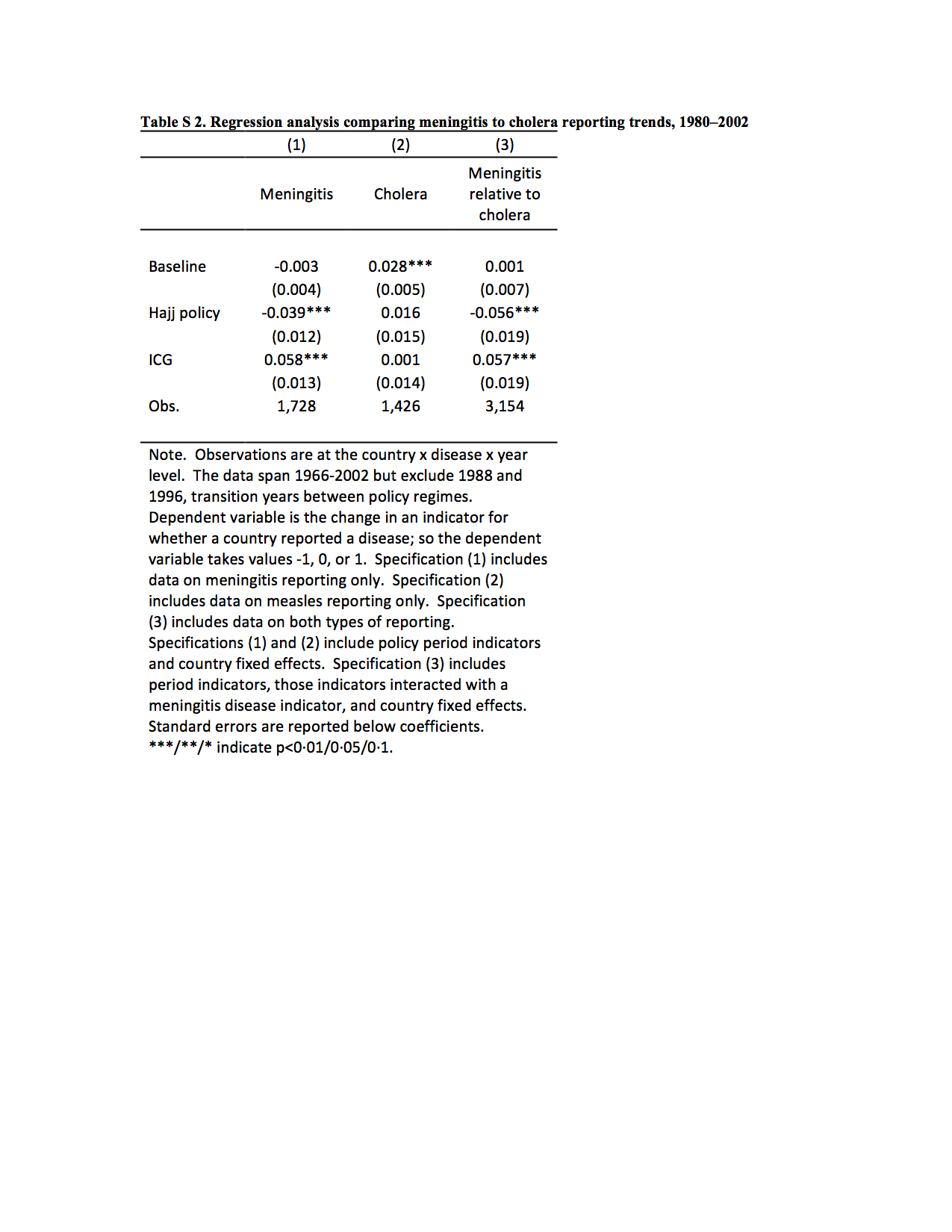

Supplement: Table S2 — Regression analysis comparing meningitis to cholera reporting trends, 1980–2002. (TIFF) [file pone.0090290.s003.tiff]

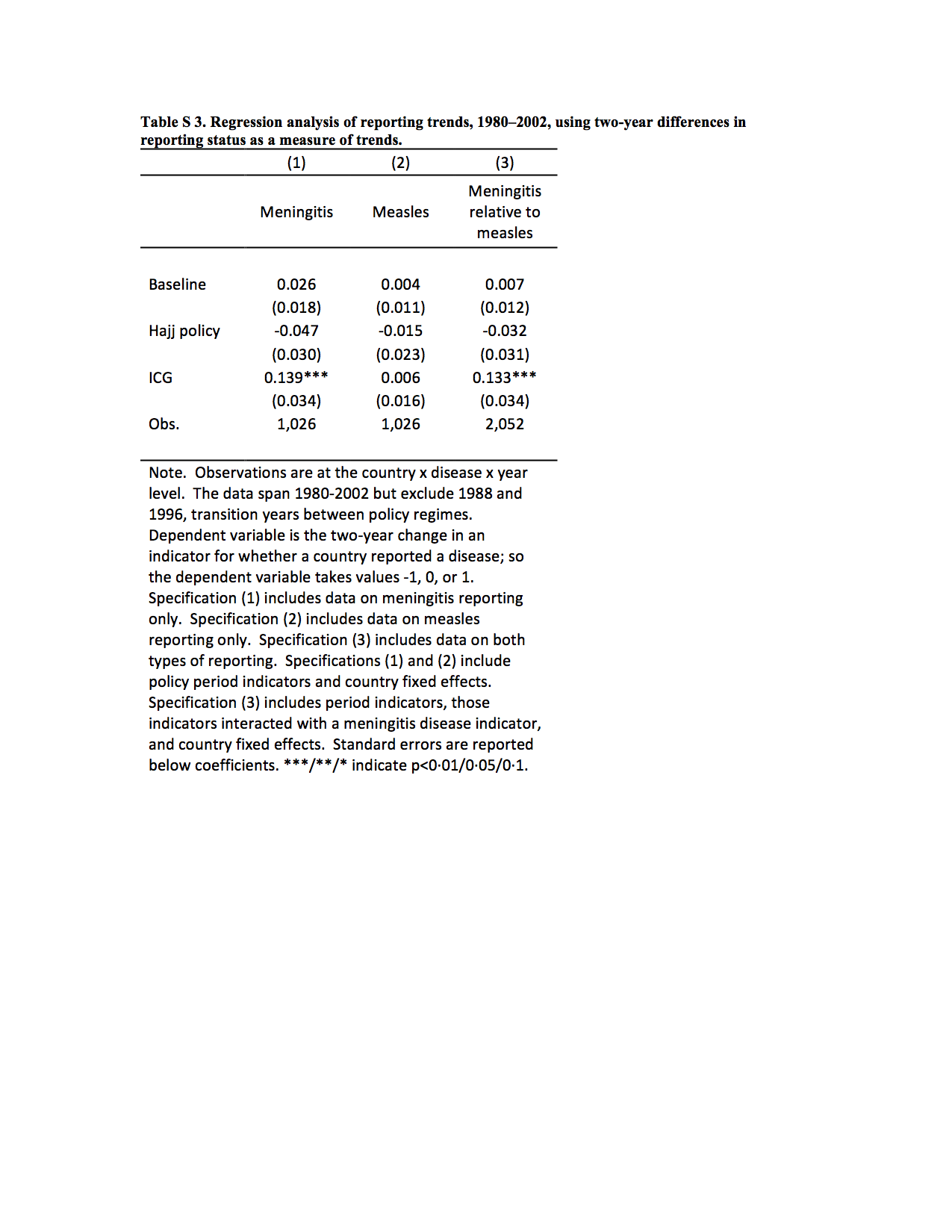

Supplement: Table S3 — Regression analysis of reporting trends, 1980–2002, using two-year differences in reporting status as a measure of trends. (TIFF) [file pone.0090290.s004.tiff]
